# A novel function of *N*-linked glycoproteins, alpha-2-HS-glycoprotein and hemopexin: Implications for small molecule compound-mediated neuroprotection

**DOI:** 10.1371/journal.pone.0186227

**Published:** 2017-10-09

**Authors:** Takuya Kanno, Kaori Yasutake, Kazunori Tanaka, Shinji Hadano, Joh-E Ikeda

**Affiliations:** 1 NGP Biomedical Research Institute, Neugen Pharma Inc., Meguro, Tokyo, Japan; 2 Department of Molecular Life Sciences, Tokai University School of Medicine, Isehara, Kanagawa, Japan; 3 The Institute of Medical Sciences, Tokai University, Isehara, Kanagawa, Japan; 4 Department of Molecular Neurology, Faculty of Medicine, Kitasato University, Sagamihara, Kanagawa, Japan; 5 Apoptosis Research Centre, Children’s Hospital of Eastern Ontario, Department of Pediatrics, Faculty of Medicine, University of Ottawa, Ottawa, Ontario, Canada; Hungarian Academy of Sciences, HUNGARY

## Abstract

Therapeutic agents to the central nervous system (CNS) need to be efficiently delivered to the target site of action at appropriate therapeutic levels. However, a limited number of effective drugs for the treatment of neurological diseases has been developed thus far. Further, the pharmacological mechanisms by which such therapeutic agents can protect neurons from cell death have not been fully understood. We have previously reported the novel small-molecule compound, 2-[mesityl(methyl)amino]-N-[4-(pyridin-2-yl)-1H-imidazol-2-yl] acetamide trihydrochloride (WN1316), as a unique neuroprotectant against oxidative injury and a highly promising remedy for the treatment of amyotrophic lateral sclerosis (ALS). One of the remarkable characteristics of WN1316 is that its efficacious doses in ALS mouse models are much less than those against oxidative injury in cultured human neuronal cells. It is also noted that the WN1316 cytoprotective activity observed in cultured cells is totally dependent upon the addition of fetal bovine serum in culture medium. These findings led us to postulate some serum factors being tightly linked to the WN1316 efficacy. In this study, we sieved through fetal bovine serum proteins and identified two *N*-linked glycoproteins, alpha-2-HS-glycoprotein (AHSG) and hemopexin (HPX), requisites to exert the WN1316 cytoprotective activity against oxidative injury in neuronal cells *in vitro*. Notably, the removal of glycan chains from these molecules did not affect the WN1316 cytoprotective activity. Thus, two glycoproteins, AHSG and HPX, represent a pivotal glycoprotein of the cytoprotective activity for WN1316, showing a concrete evidence for the novel glycan-independent function of serum glycoproteins in neuroprotective drug efficacy.

## Introduction

Oxidative stress in the central nervous system (CNS) plays a critical role in the pathogenesis of many acute as well as chronic neurological disorders, such as stroke, Alzheimer’ s disease, Parkinson’s disease, Huntington’s disease, and amyotrophic lateral sclerosis (ALS) [1]. We have previously identified the novel small molecule compound, 2-[mesityl(methyl)amino]-N-[4-(pyridin-2-yl)-1H-imidazol-2-yl] acetamide trihydrochloride (named WN1316), as a unique oxidative stress suppressor and a therapeutic candidate for the treatment of ALS [2], by employing a new combined approach for the drug screening with *in silico* drug designing and *in silico* drug screening by an algorithm of quantitative relationship between the chemical structure of the compound and its anti-oxidative neuronal cell death activity via up-regulation of neuronal apoptosis inhibitory protein (NAIP) [2–6]. However, the precise molecular as well as pharmacological mechanisms by which WN1316 protects neurons from oxidative stress through suppression of IL1-β and gliosis *in vivo* have been remained to be elucidated.

During the course of study, we found that the concentration of WN1316 in the brain after oral administration in wild-type mice was approximately three times higher than that in serum, despite the fact that almost all WN1316 molecules bound to serum proteins [2]. By contrast, the concentrations of the WN1316-sister compounds in the brain and serum after oral administration in mice were most similar [2]. It is generally noticed that the dose range of a drug required to produce *in vivo* efficacy is substantially higher than *in vitro* potency [7]. However, the efficacy of WN1316 in ALS mouse models emerged at a sub-nanomolar range, which was rather hundred times lower than its potency in cultured human neuronal cells [2]. Differences in the efficacy dose of WN1316 between *in vitro* and *in vivo* implies the presence of a mechanism by which WN1316 is preferentially enriched in the brain. Remarkably, it has been demonstrated by our preliminary study that fetal bovine serum (FBS) is a requisite for the cytoprotective activity of WN1316 *in vitro*. These led us to hypothesize that some of serum proteins closely linked to the WN1316-dependent cytoprotective activity.

In this study, we attempted to identify the biological WN1316-activating factors in FBS. We successfully identified nine candidate serum proteins by using a proteomic approach in conjunction with quantitative WN1316 cytoprotective activity analysis. Among them, two *N*-linked glycoproteins, alpha-2-HS-glycoprotein (AHSG) and hemopexin (HPX), markedly exhibited the WN1316-mediated cytoprotection against oxidative injury in differentiated neuroblastoma SH-SY5Y cells. Thus, these two glycoproteins play a key role in exerting the WN1316-mediated cytoprotective activity *in vitro*, suggesting that they are ever-unidentified possible endogenous factors implicating in the effective drug delivery to the CNS.

## Materials and methods

### Cells and reagents

Human neuroblastoma SH-SY5Y cells (CRL-2266) were obtained from the American Type Culture Collection (Manassas, VA). WN1316 was synthesized by Wakunaga Pharmaceutical Co., Ltd. (Hiroshima, Japan). The purity of the compounds is higher than 99.2%. We purchased AlamarBlue from Invitrogen (Carlsbad, CA), PNGase F, and Protein Deglycosylation Mix from New England Biolabs (Tokyo, Japan), XerumFree FBS replacement from TNCBIO (Eindhoven, Netherlands), WEPRO1240G Expression Kit from Cell-Free Sciences (Yokohama, Japan), and all-*trans* retinoic acid and Dulbecco’s modified Eagle’s medium (DMEM) from Wako Pure Chemical Industries, Ltd. (Osaka, Japan). General laboratory reagents were obtained from Nacalai Tesque (Kyoto, Japan) and Sigma (St. Louis, MO). All other laboratory reagents were from commercial sources and of analytical grade.

### Anti-oxidative stress-induced neuronal cell death (AOND) assay

SH-SY5Y cells were grown in DMEM supplemented with 10% non-heat inactivated FBS (HyClone, Thermo Fisher Scientific, Waltham, MA), 100 U/ml penicillin, and 100 μg/ml streptomycin at 37°C in 5% CO_2_. SH-SY5Y cells were seeded at a density of 0.75 × 10^4^ cells per well in 96-well plates (Primaria; BD Falcon, Franklin Lakes, NJ) for 1 day and were differentiated in the medium containing 10 μM all-*trans* retinoic acid (RA) [8] for additional 5 days. Under such culture conditions, majority of SH-SY5Y cells showed the neuron-like morphology with growing neurites. We also confirmed a significant increase in the expressions of neuron specific enolase (NSE) and neuronal nuclei (NueN) in RA-differentiated SH-SY5Y cells by Western blotting (data not shown).

WN1316 was dissolved in dimethyl sulfoxide (DMSO) to prepare 50 mM stock solution. Differentiated SH-SY5Y cells were then treated with 8 μM WN1316 for 3 h followed by 12 h of chase incubation without the compound. Cells treated with DMSO only were handled as vehicle control in this assay. Then, cells were exposed to 40 μM menadione for 4 h. Cell viability (the percentage of viable cell numbers under menadione-treated / -untreated conditions) was measured by AlamarBlue assay as described previously [3]. In this study, the WN1316-mediated cytoprotective activity was expressed as a relative value of the cell viability in WN1316-treated cells for that in DMSO-treated control. For WN1316-activating factor additive experiments, we used serum-free medium containing 10% XerumFree FBS replacement instead of 10% FBS to exclude the influence of FBS in the assay.

### Separation of active protein fractions from FBS

#### Ammonium sulfate precipitation

FBS (HyClone, 7 ml) was fractionated by addition of pulverized ammonium sulfate to make the final concentration of 50% on ice with gentle stirring for 30 min. The 0–50% ammonium sulfate precipitate (ASP_0-50%_) was collected by centrifugation at 13,000 x *g* for 30 min. The following steps were performed at 4°C unless otherwise stated.

#### HiTrap Blue HP column chromatography

ASP_0-50%_ was dissolved in approximately 1 ml of 20 mM HEPES buffer (pH 7.4), and dialyzed against 20 mM HEPES buffer (pH 7.4). After dialysis, sample (total protein amount of 42 mg) was applied to a HiTrap Blue HP column (5 ml, GE Healthcare, Tokyo, Japan) equilibrated with 20 mM HEPES buffer (pH7.4) using a manual syringe. After a 50-ml wash with 20 mM HEPES buffer (pH 7.4), the elution of proteins bound to the column was performed with a step-wise elution with 20 mM HEPES buffer (pH 7.4) containing 2 M NaCl, and 1 ml of fractions were collected. Aliquot of fractions from the column unbound (Blue pass) and bound elutes (Blue elute) were subjected to the AOND assay and protein content analysis (S1 Fig).

#### Bio-Scale Mini CHT Type I column chromatography

The active pass-through fraction (total protein amount of 26 mg) from HiTrap Blue HP column was dialyzed against 5 mM phosphate buffer (starting buffer, pH 6.5) and loaded onto a Bio-Scale Mini CHT Type I column (5 ml, Bio-Rad, Hercules, CA) equilibrated with starting buffer using an AKTA Prime Plus system (GE Healthcare). After a 50-ml wash with starting buffer, protein was eluted with a linear gradient of 0–3 M NaCl in starting buffer (100 ml) at a flow rate of 0.5 ml/min, followed by the elution with 50 ml of 300 mM phosphate buffer (pH 6.5), and 1ml of fractions were collected. Aliquots of fractions were subjected to the AOND assay and protein content analysis. The fraction numbers 6 to 14 (named as W1) from washing fraction, 61 to 62 (named as E1) from elution fraction of 0–3 M NaCl in starting buffer, and 119 to 122 (named as C1) from elution fraction of 300 mM phosphate buffer (pH 6.5) were pooled, respectively. Each pooled fraction was concentrated by an Amicon ultra-15 (Millipore, Bedford, MA), and stored at 4°C until use.

Protein concentration was quantified by Pierce 660 nm Protein Assay kit (Thermo Fisher Scientific) using bovine serum albumin (BSA) as a standard. In this study, one unit of the WN1316-activating factor activity was defined as the amount of protein required to increase 1% cell viability when using FBS.

### Proteomics analysis

In this analysis, fraction E1, C1, and W1 from Bio-Scale Mini CHT Type I column chromatography were rendered to two-dimensional polyacrylamide gel electrophoresis (2DE). These proteomics analyses were conducted by Towa Environment Science Co., Ltd. (Hiroshima, Japan). Briefly, the sample (300 μg) was loaded onto reswollen gel strip with immobilized pH gradient (IPG) (pH 4 to 7, 24 cm long, GE Healthcare) at 20°C for overnight. The proteins were then separated by isoelectric focusing (IEF) using a CoolPhoreStar IPG-IEF Type-P (Anatech, Tokyo, Japan) at 20°C and focused with the following program: 500 V for 1 min, 500 to 3,500 V for 90 min, and 3,500 V for 14.5 h. After completion of IEF, the strips were sodium dodecyl sulfate (SDS) equilibrated, reduced and alkylated with dithiothreitol and iodoacetamide, followed by separation on a 9–18% gradient SDS-polyacrylamide gel electrophoresis (SDS-PAGE) gels (26 x 22 cm, Laboratory-made) using the Anderson ISO-DALT electrophoresis system (Hoefer) at 80 V constant for 16 h. All the experiments were run in quadruplicate for each sample to ensure reproducibility. The gels were stained with SYPRO Ruby protein gel stain (Molecular Probes) and scanned with Molecular Imager FX (Bio-Rad). Image analysis and spot matching were performed using the ImageMaster 2D Platinum (GE Healthcare). The quantitative level of each protein spot was determined by the relative volume of each spot in the gel and expressed as %Volume (spot volume/Σ volumes of all spots). Protein spots were excised from the 2DE and digested with trypsin (37°C, 16 h). The resulting peptides were concentrated and desalted using Zip Tip C18 (Millipore), and mixed with α-cyano-4-hydroxycinnamic acid (Wako) and spotted into wells of a MTP Anchorchip 600/384 (Bruker daltonics). Mass spectra were acquired in reflector positive ion mode using an Ultraflex MALDI-TOF/TOF mass spectrometer (MS) (Bruker daltonics). Selected peptides were fragmented in the second dimension, and the proteins were identified by mass searches in the NCBInr database using the MS/MS ion search software Mascot (Matrix Science, http://www.matrixscience.com). The identified proteins were analyzed its properties and functions by using the the UniProt Knowledgebase (UniPortKB) database (http://www.uniprot.org), which is a central hub of protein knowledge by providing a unified view of protein sequence and functional information [9].

### Separation of *N*-linked glycoproteins using a Con A lectin column

FBS was buffer-exchanged against 20 mM HEPES, 0.5 M NaCl, 1 mM MnCl_2_, 1 mM CaCl_2_ (binding buffer, pH 7.4) by Zeba Spin Desalting Columns (Thermo Fisher Scientific). The buffer-exchanged FBS (1 ml) was loaded onto a HiTrap Con A 4B column (1 ml, GE Healthcare) equilibrated with binding buffer using a manual syringe. After a 25-ml wash with binding buffer, the elution of proteins bound to the column was performed with 25 ml of 20 mM HEPES, 0.5 M NaCl, 0.5 M methyl-α-D-glucoside (pH 7.4), and 0.5 ml of fractions were collected. Aliquot of fractions from column unbound (Con A pass) and bound elutes (Con A elute) were subjected to the AOND assay and protein concentration analysis.

### Deglycosylation of proteins

Removal of glycans from target proteins in Blue pass fraction was carried out by PNGase F, which cleaved *N*-linked oligosaccharides from glycoproteins [10], and Protein Deglycosylation Mix (EnzMix) containing PNGase F, *O*-glycosidase, neuraminidase (sialidase), β1–4 galactosidase, and β-*N*-acetylglucosaminidase, which removed both *N*-glycans and some *O*-glycans [11]. Under non-denaturing conditions, Blue pass fraction (200 μg) was incubated with or without PNGase F (1 unit) or EnzMix (1 unit) in 50 μl of 50 mM sodium phosphate (pH 7.5) at 37°C for 16 h. The molecular masses and homogeneities of the proteins, with or without the enzyme treatment, were analyzed by Western blotting with anti-AHSG antibody as a marker of glycoproteins.

### Western blotting

Proteins (0.2 μg/lane) were resolved by SDS-PAGE on a 5–20% gradient gel (ATTO, Tokyo, Japan) and transferred onto polyvinylidene difluoride membrane (Bio-Rad). Membranes were blocked with Blocking-One reagent (Nacalai Tesque) for 1 h at room temperature and then incubated with the anti-AHSG antibody (1:1,000 dilution; ab112528; Abcam, Cambridge, UK) in Can Get Signal Solution 1 (TOYOBO, Osaka, Japan) overnight at 4°C. The membrane was washed with TBS-T (50 mM Tris-HCl, pH 7.4, 150 mM NaCl, 0.1% Tween 20) three times and incubated for 1 h at room temperature with horseradish peroxidase-conjugated secondary anti-rabbit IgG (#NA934, GE Healthcare) in Can Get Signal Solution 2 (TOYOBO). The membrane was washed as described above, and then signals were detected using Immobilon Western HRP Substrate (Millipore) and X-ray film.

### Plasmid construction

For the synthesis of glutathione S-transferase (GST) fusion proteins by the wheat germ cell-free system, three human cDNAs, *AHSG* (FXC03806), *SERPINA1* (FXC03481) and *RBP4* (FXC03871), were obtained from Flexi ORF clone (Promega, Madison, WI) and were amplified by PCR in a GeneAmp PCR system 9700 (Applied Biosystems, Tokyo) with PrimeSTAR Max DNA Polymerase (TaKaRa) according to the manufacturer's instructions. Specific primer sets were as follows: *AHSG*, 5′-AAAACTAGTATGAAGTCCCTCGTCCTGCTCCTTTG-3' and 5′-ATGCTAGTTATTGCTCAGCGG-3'; *SERPINA1*, 5′-AAAACTAGTATGCCGTCTTCTGTCTCGTGGGGCA-3' and 5′-ATGCTAGTTATTGCTCAGCGG-3'; *RBP4*, 5′-AAAACTAGTATGAAGTGGGTGTGGGCGCTCTTGC-3' and 5′-AAACTCGAGTTAAACCAAAAGGTTTCTTTCTGAT-3'. Four human cDNAs, *A1BG*, *CFB*, *ITIH4* and *HPX* were amplified from Human Total RNA Master Panel II (Clontech, Palo Alto, CA) by RT-PCR with iScript cDNA synthesis kit (Bio-Rad) and PrimeSTAR Max DNA Polymerase (TaKaRa), following the manufacturer's instructions. PCR primers specific to each cDNA were as follows: *A1BG*, 5′-ATGTCCATGCTCGTGGTCTTTCTC-3' and 5′-AAAAGTCGACTCAGCTTTCTGCCACCAGGAGCTC3'; *CFB*, 5′-ATGGGGAGCAATCTCAGCCCCCAA-3' and 5′-AAAAGTCGACTCATAGAAAACCCAAATCCTCATC-3'; and *HPX*, 5′-ATGGCTAGGGTACTGGGAGCACCC-3' and 5′-AAAAGTCGACCTAGATGTGGGCTACAGCGCATAT-3'. The *ITIH4* primers were designed to amplify the 70-kDa mature form of human ITIH4 (residues 85–1983) [12] and they were as follows: 5′-AAAACTAGTATGGAAAAGAATGGCATCGACATCTA-3' and 5′-AAAAGTCGACCTACAGCTCCACAGACCAGCAGGA-3'. Amplified fragments were digested with *Sal*I (*AHSG*, *SERPINA1*, *A1BG*, *CFB*, *ITIH4* and *HPX*) or *Xho*I (RBP4), and then cloned into the *Sma*I-*Sal*I or *Sma*I-*Xho*I sites of pEGST expression vector [13], respectively. The resulting plasmids were designated pEGST-AHSG, pEGST-SERPINA1, pEGST-A1BG, pEGST-CFB, pEGST-ITIH4, pEGST-HPX, and pEGST-RBP4, respectively.

### Cell-free protein synthesis

Wheat germ cell-free translational system is most suitable for the expression of unmodified full length eukaryotic proteins from plant and animal sources, and is capable of producing proteins with proper folding [14–16]. Cell-free protein synthesis was performed with WEPRO1240G Expression Kit according to the manufacturer's instructions. GST fusion proteins synthesized by the cell-free system were purified through a glutathione-Sepharose 4B column (GE Healthcare) according to the instructions of the manufacturer.

### Statistical analysis

Data in this study were presented as mean ± standard deviation (SD). Statistical analyses were conducted using PRISM 5 (GraphPad). Statistical significance was evaluated by ANOVA (analysis of variance) followed by Dunnett’s method for multiple comparisons between groups. We considered *p*-values < 0.01 to be statistically significance.

## Results

### Protein factors in FBS exert WN1316-mediated cytoprotective activity

**Fig 1:**
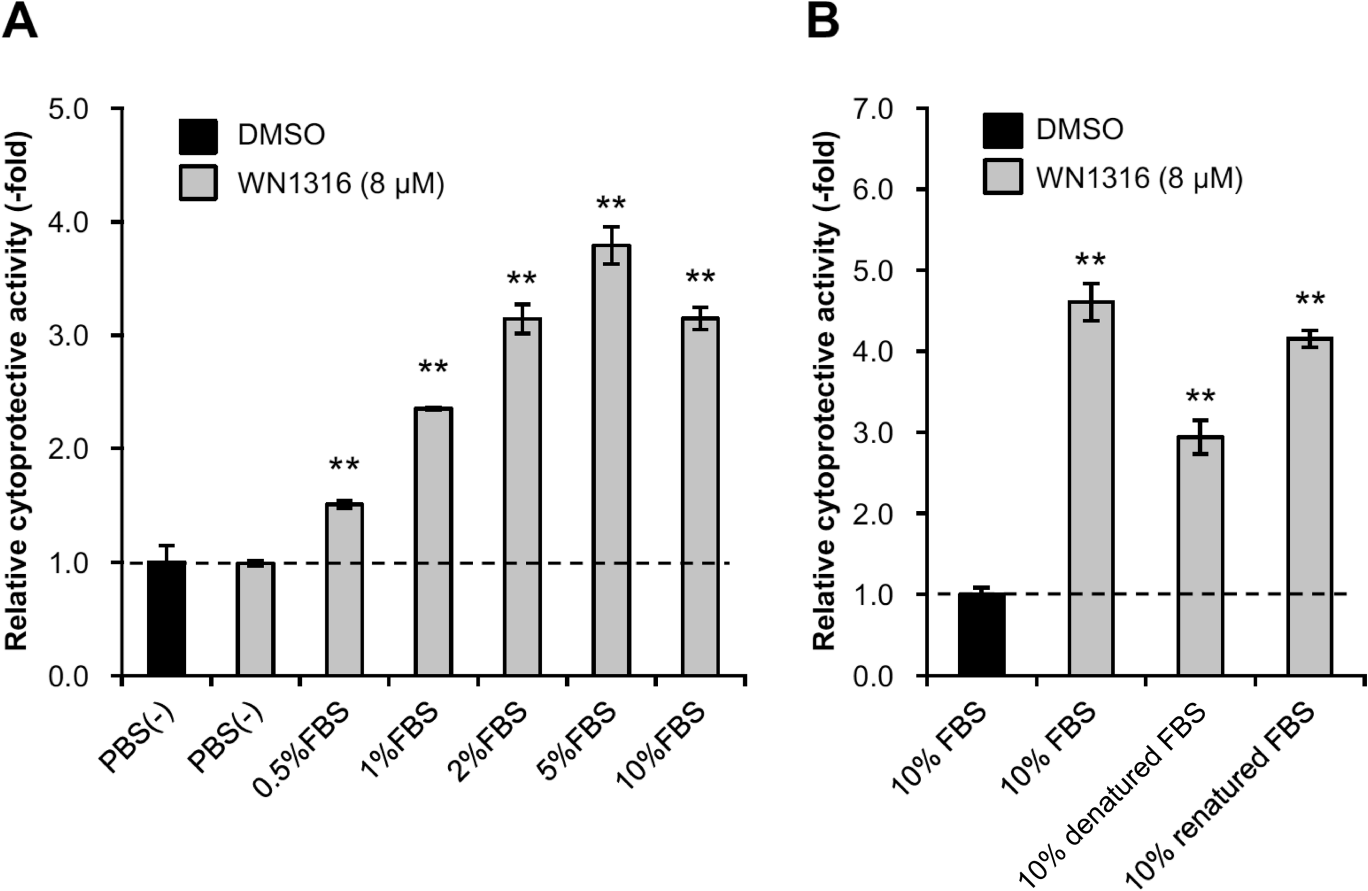
Protein factors in FBS exert WN1316-mediated cytoprotective activity. (A) Effect of FBS on WN1316-induced cytoprotection. Differentiated SH-SY5Y cells were treated with 8 μM WN1316 or DMSO for 3 h followed by 12 h of chase incubation without the compound, and then exposed to 40 μM menadione for 4 h. The cell viability was measured by AlamarBlue assay, and was expressed as a relative value (relative cytoprotective activity; -fold) of the WN1316-treated samples for vehicle control (DMSO) set as 1. Data are expressed as mean ± SD (n = 4). Statistical significance was evaluated by one-way ANOVA (*p*<0.0001) followed by Dunnett’s *post hoc* test compared with DMSO-treated control (**p<0.001). (B) Effect of heat-denatured FBS on the anti-oxidative stress activity of WN1316. Heat denaturation of FBS was performed at 65°C for 30 min and chilled ice-cold (denatured FBS), and heat denatured FBS was renatured by the incubation at 4°C for 16 h (renatured FBS). Differentiated SH-SY5Y cells were incubated with 8 μM WN1316 or DMSO for 3 h in DMEM supplemented with 10% FBS, 10% denatured FBS, or 10% renatured FBS, followed by 12 h of chase incubation without the compound, and then treated with 40 μM menadione for 4 h. The cell viability was calculated by AlamarBlue assay, and was expressed as a relative value (relative cytoprotective activity; -fold) of the WN1316-treated samples for vehicle control (DMSO) set as 1. Data are expressed as mean ± SD (n = 4). Statistical significance was evaluated by one-way ANOVA (*p*<0.0001) followed by Dunnett’s *post hoc* test compared with DMSO-treated control (**p<0.001).

In our preliminary study, we found that FBS was essential to exhibit the cytoprotective activity of WN1316 *in vitro* (data not shown). To verify this, we investigated whether FBS affected the cytoprotective activity of WN1316 by AOND assay. The WN1316-meidated cytoprotective activity was observed in a FBS dose-dependent manner, but not in serum-free conditions (Fig 1A). The results suggest that actual component in FBS is vital for the cytoprotective activity of WN1316.

To investigate the nature of the component, we next analyzed whether the WN1316 cytoprotective activity was affected by the heat-denature treatment of FBS. In this study, we used mild conditions (65°C), which was higher than those in heat-inactivation of FBS (56°C), to avoid the heat-induced protein precipitation. The WN1316 cytoprotective activity was decreased under the cell culture conditions with heat-denatured FBS, while it was partially recovered by the renatured FBS (Fig 1B). These results indicate that thermolabile protein(s) plays a pivotal role in the WN1316-mediated cytoprotection. Here, we designated these components as WN1316-activating factors. Since it was revealed that the effective concentrations of FBS ranged from 1 to 5% (Fig 1A), we used 2% of FBS, as a positive control, in all the following assays for the WN1316-mediated cytoprotective activity.

### Separation of WN1316-activating factors from FBS

**Fig 2:**
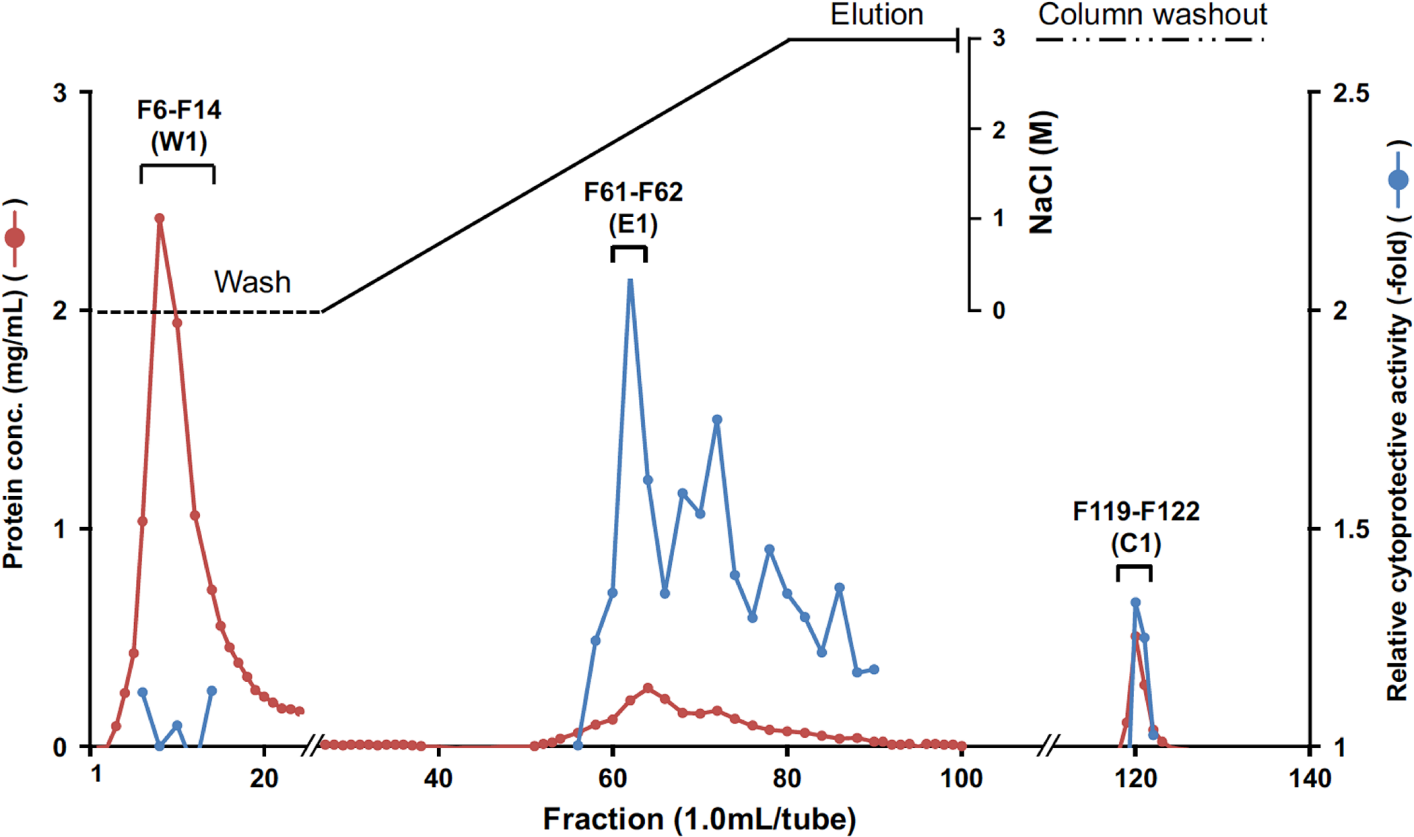
Fractionation of WN1316-activating factor by Bio-Scale Mini CHT Type I chromatography. The active elution sample from a HiTrap Blue HP column was applied to a Bio-Scale Mini CHT Type I column equilibrated with 5 mM phosphate buffer (starting buffer, pH 6.5) at a flow rate of 0.5 ml/min. The column was washed with 50 ml of starting buffer. Proteins were eluted by a linear gradient of NaCl from 0 to 3 M in starting buffer (100 ml), and a final column washout was performed with 50 ml of 300 mM phosphate buffer (pH 6.5). One-ml fractions were collected and 4-μl aliquots assayed for the WN1316-mediated cytoprotective activity. Differentiated SH-SY5Y cells were pretreated with 8 μM WN1316 or DMSO in the presence of each fraction (4 μl/well) for 3 h followed by 12 h of chase incubation without the compound, and then exposed to 40 μM menadione for 4 h. The cell viability was calculated by AlamarBlue assay, and was expressed as a relative value (relative cytoprotective activity; -fold) of the WN1316-treated samples for vehicle control (DMSO) set as 1. The active fractions were pooled.

To identify the WN1316-activating factors, we sieved proteins, which were not associated with the WN1316 cytoprotective activity, out of FBS. Our preliminary study showed that bovine serum albumin (BSA), a major serum component, had no relation to the WN1316 cytoprotective activity (data not shown). Therefore, we firstly removed BSA from FBS by 50% saturated ammonium sulfate precipitation and following HiTrap Blue HP chromatography. The WN1316 cytoprotective activity was concentrated in the ammonium sulfate fraction and recovered in the pass-through fraction (Blue pass) of HiTrap Blue HP column (Table 1, S1 Fig). Subsequently, the Blue pass fraction was subjected to a Bio-Scale CHT Type I hydroxyapatite affinity chromatography (Fig 2). An approximately 80% of the loaded protein passed through the column and was found no WN1316 cytoprotective activity. Thus, fraction numbers from 6 to 14 were pooled as a non-activity fraction W1. Although the WN1316 cytoprotective activity was broadly eluted out, the highest activity was eluted in fraction numbers 61 and 62, and they are pooled as a high-specific activity fraction E1. Residual WN1316 cytoprotective activity in the wash out fractions (fraction numbers 119 to 122) were also pooled as a low-specific activity fraction C1.

**Table 1:** Purification of WN1316-activating factors.

Purification step	Total protein (mg)	Total activity (units)*	Specific activity (units/mg)	Purification (-fold)	Yield (%)
FBS	161	1343	8.34	1.0	100
50% ammonium sulfate precipitation	42	856	20.6	2.5	64
HiTrap Blue HP pass fraction	26	692	26.6	3.2	51
Bio-Scale Mini CHT Type I fraction E1	0.21	228	979	117	17

* One unit of the WN1316-activating factor activity is defined as the amount of the protein required to increase 1% cell viability when using FBS.

The specific activity of each purification step, 50% saturated ammonium sulfate precipitation fraction, Blue pass fraction, and high-specific activity fraction E1 on Bio-Scale CHT Type I hydroxyapatite affinity chromatography, was 20.6, 26.6, and 979 units/mg, respectively (Table 1). Through these purification steps, WN1316-activating factor was concentrated 117-fold in E1 with a final yielded of 17%.

### Identification of WN1316-activating factor candidates by proteomics analysis

**Fig 3:**
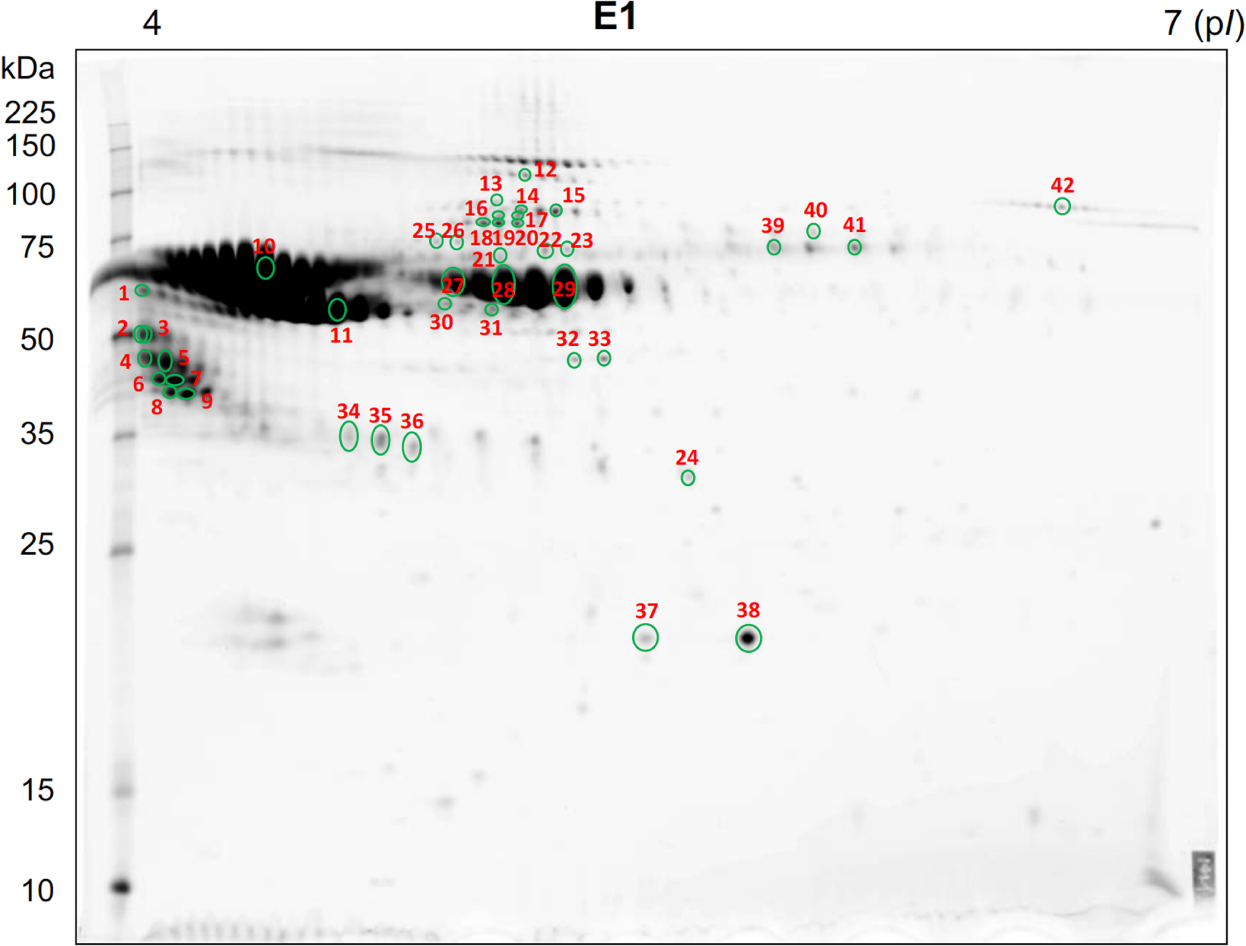
Representative 2DE gel image of high WN1316 active E1 fraction separated by the Bio-Scale Mini CHT Type I column. Proteins (300 μg) were separated by isoelectric focusing using Immobiline DryStrip of pH 4–7, followed by 9–18% gradient SDS-PAGE gels. Proteins were visualized by SYPRO Ruby staining. Proteins were identified by MALDI-TOF/TOF mass spectrometry. The identified spots are numbered and labeled with circles. Spot numbers refer to numbers in Table 2.

To identify WN1316-activating factor, the high-specific activity fraction E1 was further sieved and analyzed by a proteomic approach based on 2DE/MS, while non-activity fraction W1 and low-specific activity fraction C1 were used as references for this proteomics analysis (S2 Fig). The protein spots in the 2DE gel were classified into 5 groups (S2 Fig). We sorted the spots for further analysis using the following two step ways: Step 1, choosing the E1 specific spots by comparing %volumes of the spots between E1 and the reference W1; and Step 2, selecting out the E1-increased protein spots by comparing %volumes of the spots between E1 spots chosen in Step 1 and the reference C1. As a result, 104 spots were identified in Step 1 as E1 specific signals, and in the following Step 2, 93 spots were selected as E1 increasing signals. Among these spots, 42 spots satisfied with the criteria of sufficient amounts for MS analysis (Fig 3 and Table 2). MS/MS ion search using MALDI-TOF/TOF mass spectrometry classified all the identified proteins, which were multiple spots with different isoelectric points and molecular weights, into 9 clusters; alpha-2-HS-glycoprotein (AHSG), retinol-binding protein 4 (RBP4), hemopexin (HPX), and transferrin (TF), which are related to the transport system; alpha-1-antiproteinase (SERPINA1), a protease inhibitor; gelsolin, which is involved in actin filament severing; complement factor B (CFB), a control factor involved in complement activation; inter-alpha-trypsin inhibitor heavy chain H4 (ITIH4), and anti-inflammatory protein; alpha-1B-glycoprotein (A1BG), glycoprotein with unknown function.

**Table 2:** Profiles of proteins identified by two-dimensional polyacrylamide gel electrophoresis and mass spectrometry.

Cluster	Spot number	GI number	Protein name	Deduced molecular weight	Deduced isoelectric point	Deduced N-type sugar	Function
1	1, 2, 3, 4, 5, 6, 7, 8, 9, 10, 11	27806751	Alpha-2-HS-glycoprotein (AHSG)	38,419	5.26	Yes	Endocytosis
2	12, 13, 14, 15, 16, 17, 18, 19, 20, 21, 22, 23, 24	59857769	Inter-alpha-trypsin inhibitor heavy chain H4 (ITIH4)	101,510	6.25	Yes	anti-inflammatory protein
3	25, 26	114053019	Alpha-1B-glycoprotein (A1BG)	53,554	5.30	Yes	Unknown
4	27, 28, 29, 30, 31	27806941	Alpha-1-antiproteinase (SERPINA1)	46,104	6.05	Yes	Protease inhibitor
5	32, 33	77736201	Gelsolin	80,731	5.54	No	Regulator of actin filament severing
6	34, 35, 36	95147674	Complement factor B (CFB)	85,366	7.87	Yes	Component of the alternative pathway of complement activation
7	37, 38	132403	Retinol-binding protein 4 (RBP4)	21,069	5.44	No	Transport
8	39, 40, 41	77736171	Hemopexin (HPX)	52,209	7.90	Yes	Transport
9	42	296490966	Transferrin (TF)	69,149	8.29	Yes	Transport

GI number is the MASCOT result of MALDI-TOF/TOF-MS searched from the NCBInr database (http://www.ncbi.nlm.nih.gov).

Determination of theoretical molecular weight and isoelectric point is performed by using ExPASy (http://web.expasy.org/compute_pi)

### *N*-linked glycoproteins exert WN1316-mediated cytoprotective activity

**Fig 4:**
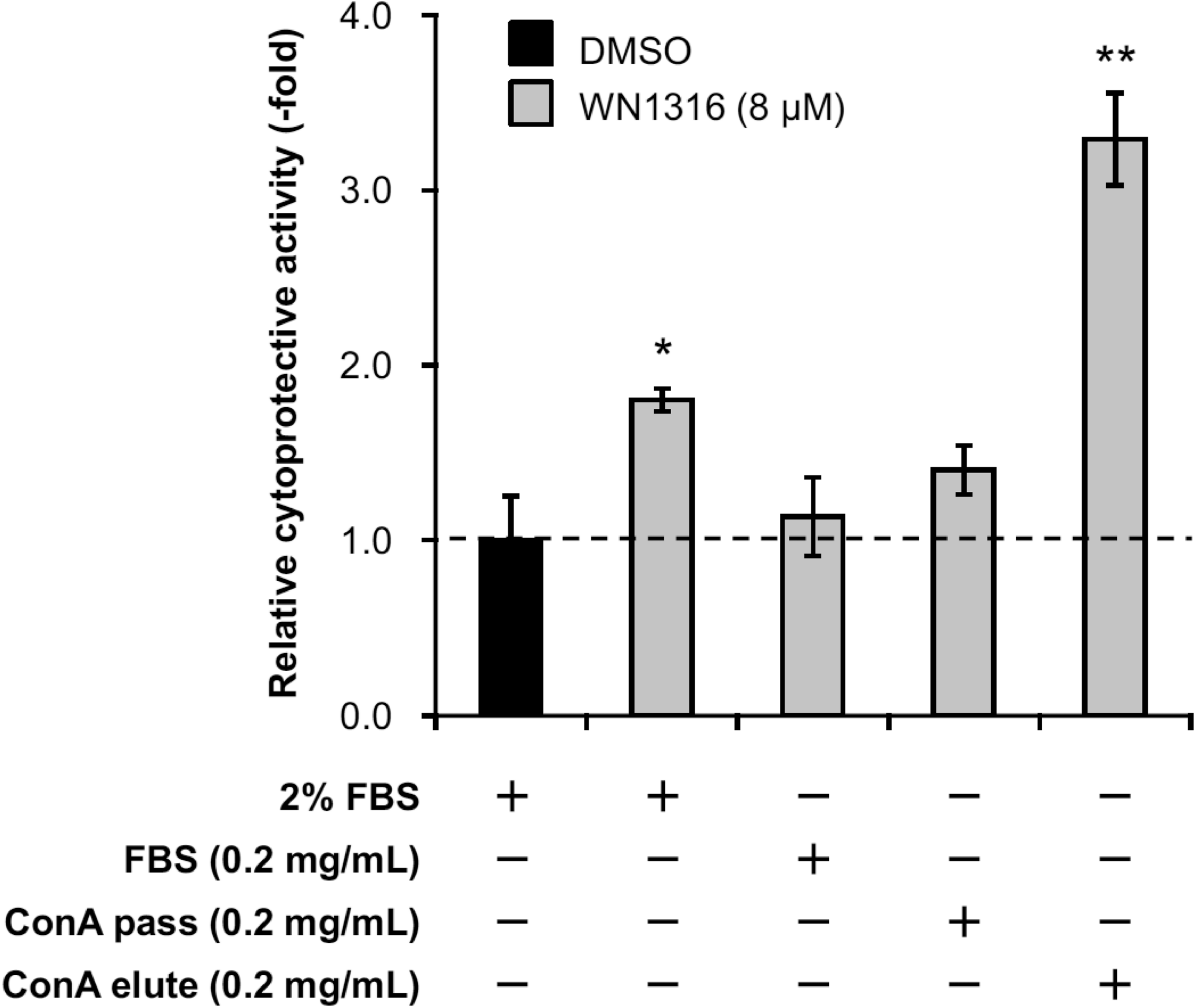
*N*-linked glycoproteins exert WN1316-mediated cytoprotective activity. Differentiated SH-SY5Y cells were pretreated with 8 μM WN1316 in DMEM containing 0.2 mg/ml FBS, Con A pass, or Con A elute for 3 h followed by 12 h of chase incubation without the compound, and then exposed to 40 μM menadione for 4 h. As control experiments, differentiated SH-SY5Y cells were treated with 8 μM WN1316 (as a positive control) or DMSO (as a vehicle control) in DMEM supplemented with 2% FBS (corresponds to a protein concentration of approximately 0.5 mg/ml). The cell viability was calculated by AlamarBlue, and was expressed as a relative value (relative cytoprotective activity; -fold) of the WN1316-treated samples for vehicle control (DMSO) set as 1. Data are expressed as mean ± SD (n = 4). Statistical significance was evaluated by one-way ANOVA (*p*<0.0001) followed by Dunnett’s *post hoc* test compared with DMSO-treated control (**p<0.001, *p<0.01).

We searched the characteristics of WN1316-activating factor candidates with the UniProtKB database, and found that seven out of them, except for gelsolin and RBP4, were glycoproteins with potential *N*-glycosylation sites. To investigate whether WN1316-activating factors are glycoproteins, we separated *N*-linked glycoproteins in FBS by using Con A lectin column and measured its WN1316-mediated cytoprotective activity. The Con A-elute as a *N*-linked glycoprotein fraction, but not the Con A pass-through as a non-glycoprotein fraction, showed the WN1316-mediated cytoprotective activity (Fig 4). These results suggest that WN1316-activating factors are *N*-linked glycoproteins. Thus, non-glycoproteins RBP4 and gelsolin were excluded from candidates.

### Deglycosylation does not affect WN1316-mediated cytoprotective activity

**Fig 5:**
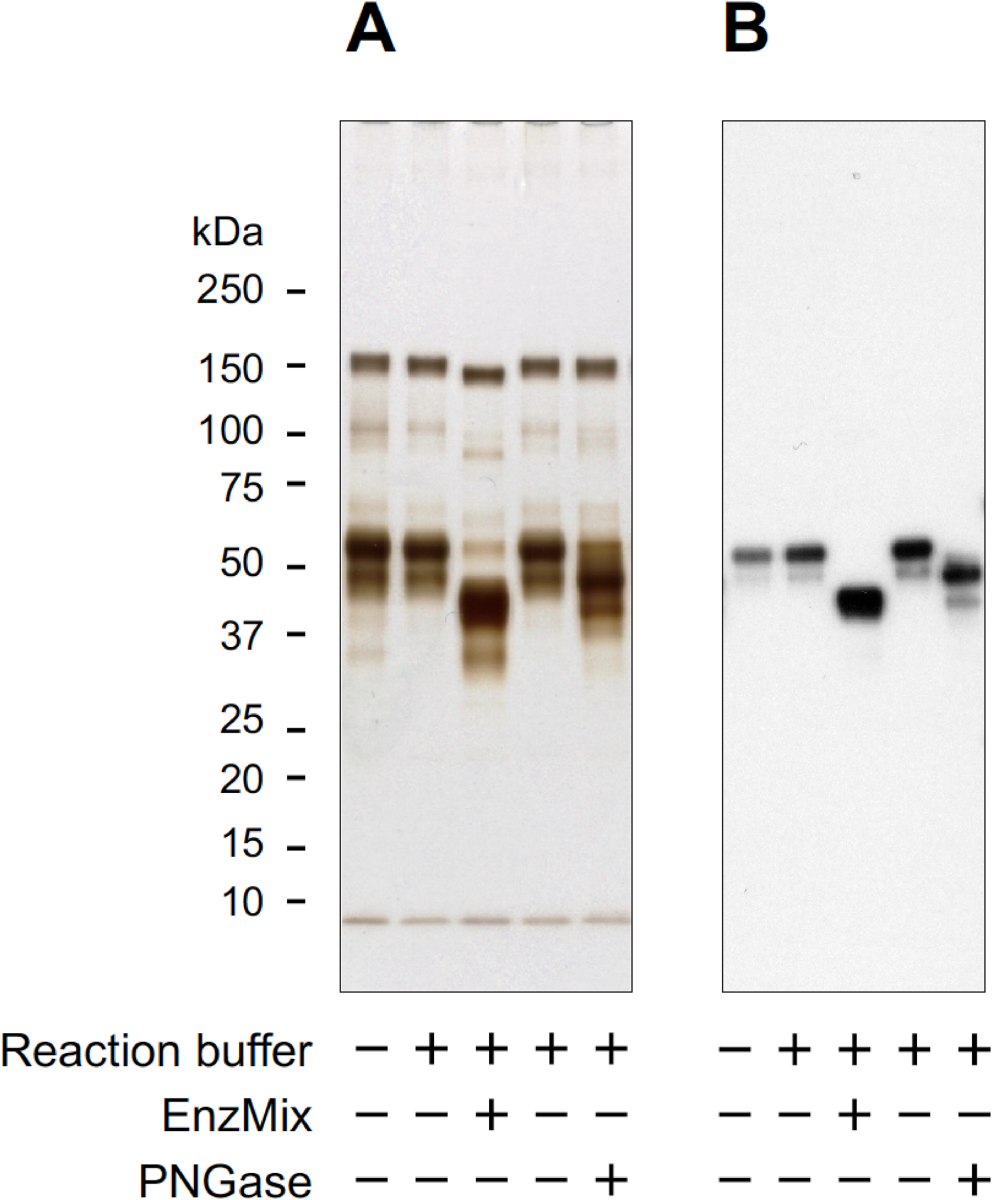
Silver staining and Western blot analysis of Blue pass fraction treated with or without glycosidase. Blue pass fraction was treated with or without EnzMix (removal *N*- and *O*-glycans) and PNGaseF (removal of *N*-glycans) at 37°C for 16 h. The molecular masses and homogeneities of the proteins were analyzed by SDS-PAGE (A) and Western blotting with anti-AHSG antibody as a glycoprotein standard (B). Proteins were visualized by silver staining using a Silver Stain Kit Wako (Wako).

**Fig 6:**
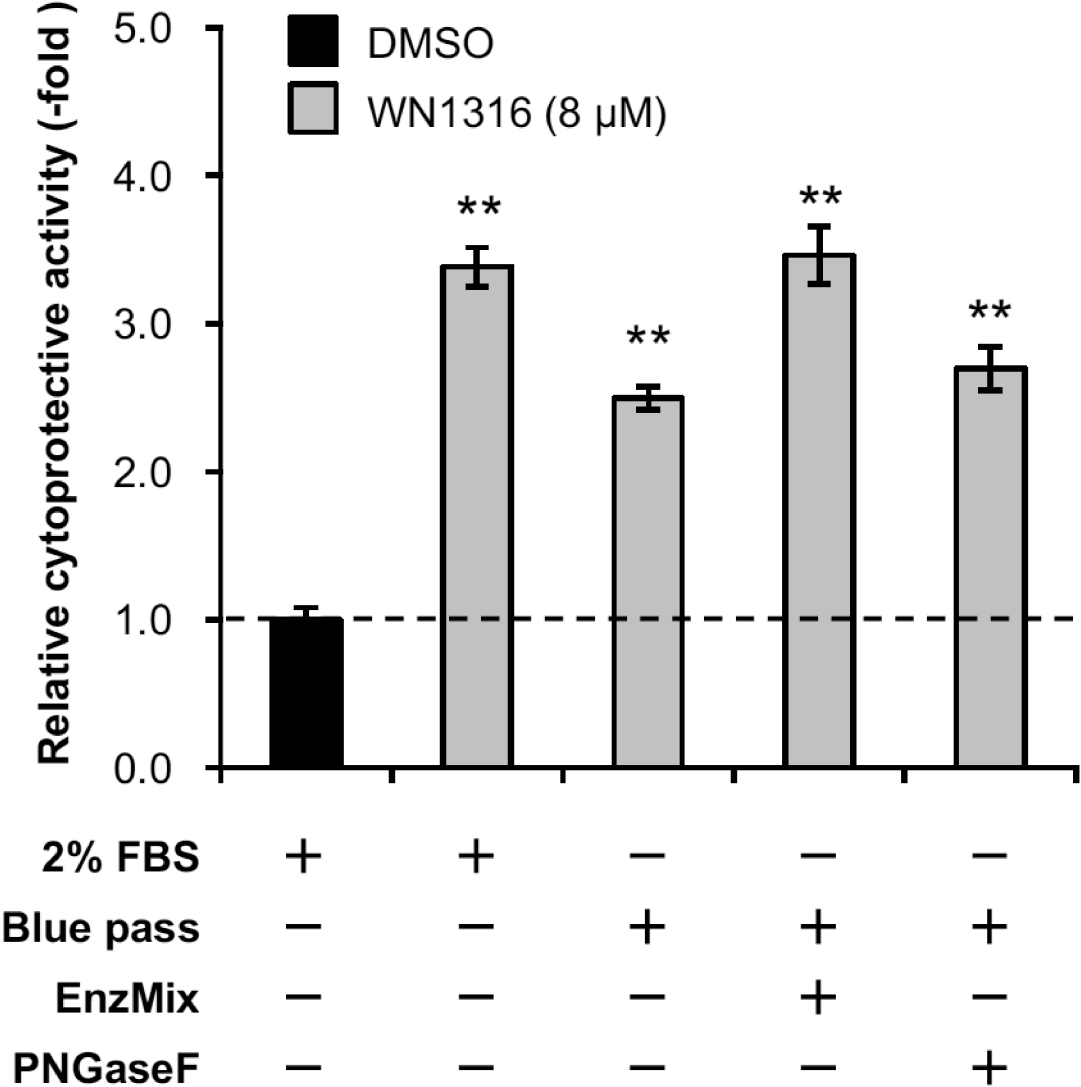
Deglycosylation does not affect WN1316-mediated cytoprotective activity. Differentiated SH-SY5Y cells were pretreated with 8 μM WN1316 in DMEM containing 0.28 mg/ml Blue pass treated with or without EnzMix and PNGaseF for 3 h followed by 12 h of chase incubation without the compound, and then exposed to 40 μM menadione for 4 h. As control experiments, differentiated SH-SY5Y cells were treated with 8 μM WN1316 (as a positive control) or DMSO (as a vehicle control) in DMEM supplemented with 2% FBS (corresponds to a protein concentration of approximately 0.5 mg/ml). The cell viability was calculated by AlamarBlue, and was expressed as a relative value (relative cytoprotective activity; -fold) of the WN1316-treated samples for vehicle control (DMSO) set as 1. Data are expressed as mean ± SD (n = 4). Statistical significance was evaluated by one-way ANOVA (*p*<0.0001) followed by Dunnett’s *post hoc* test compared with DMSO-treated control (**p<0.001).

To confirm whether the glycan chains on target proteins were associated with the WN1316-mediated cytoprotective activity, we carried out AOND assay using deglycosylation-treated FBS. The glycan chains on the glycoproteins were enzymatically digested by glycosidases. The deglycosylation was validated by SDS-PAGE and Western blotting with the commercially available antibody against AHSG as a marker of glycoproteins in FBS. We observed the differences in the protein migration patterns from FBS by the treatment with or without glycosidases (Fig 5A). We also confirmed that the AHSG signals after the treatment with glycosidases were shifted to lower molecular weight compared to non-treated control (Fig 5B). Intriguingly, WN1316-mediated cytoprotective activity was not affected by the removal of glycans on serum proteins when compared to non-treated control (Fig 6). This suggests that the sugar chains conjugated on WN1316-activating factors are not necessary for the WN1316-mediated anti-oxidative stress function *in vitro*.

### Preparation of human homologues for WN1316-activating factor candidates

The final goal of our research is to obtain the human-derived endogenous WN1316-activating factor for elucidating drug efficacy mechanism of WN1316 in human. As shown in Fig 6, since glycan chains play no major role in the WN1316 cytoprotective activity *in vitro*, we sought to evaluate the WN1316-mediated cytoprotective activity by using human homologs for the WN1316-activating factor candidates.

**Fig 7:**
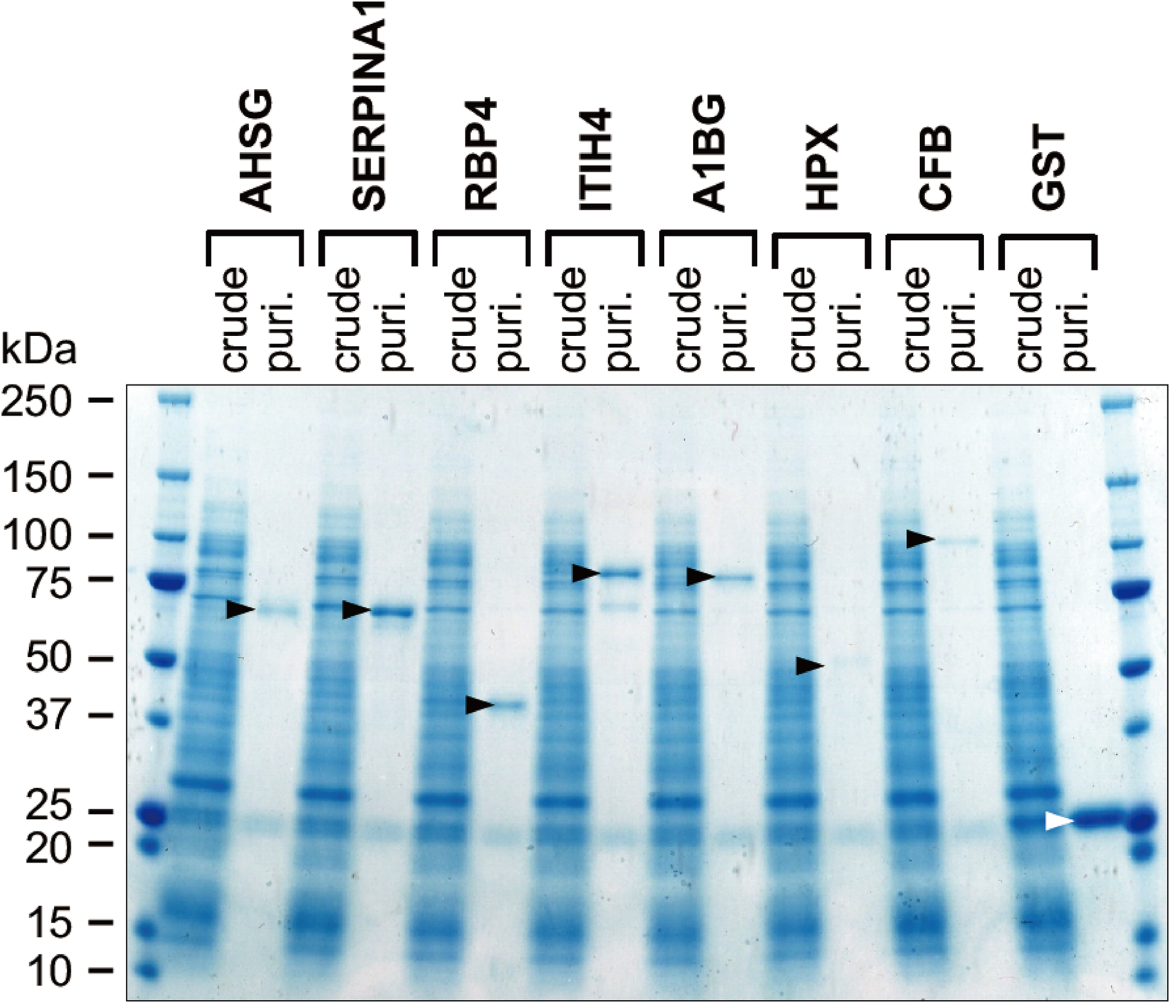
SDS-PAGE profiles of human WN1316-activating factor candidates synthesized in the cell-free system. The reaction mixture of GST protein synthesis (crude) and the GST protein after glutathione-Sepharose 4B column purification (puri.) were resolved on a 5–20% gradient SDS–PAGE gel, and gel was stained with Coomassie blue. *Black arrowheads* indicate synthesized GST fusion WN1316-activating factors. *White arrowhead* indicates GST used as a negative control.

We amplified the cDNAs of human homologue corresponding to 7 bovine WN1316-activating factor candidates (AHSG, SERPINA1, RBP4, ITIH4, A1BG, CFB, and HPX) and generated the cell-free expression constructs. Non-glycoprotein RBP4 was used as a negative control for AOND assay. Each GST-fusion protein synthesized by the wheat germ cell-free translation system was purified by a glutathione-Sepharose 4B column and analyzed the molecular masses by SDS-PAGE. We confirmed that sizes of GST-AHSG, GST-SERPINA1, GST-RBP4, GST-ITIH4, GST-A1BG, GST-CFB, and GST-HPX were 67, 74, 50, 98, 81, 56, and 113 kDa, respectively, as expected (Fig 7). We also utilized the commercially available human TF.

### Two *N*-linked glycoproteins, alpha-2-HS-glycoprotein and hemopexin, are identified as WN1316-activating factors

**Fig 8:**
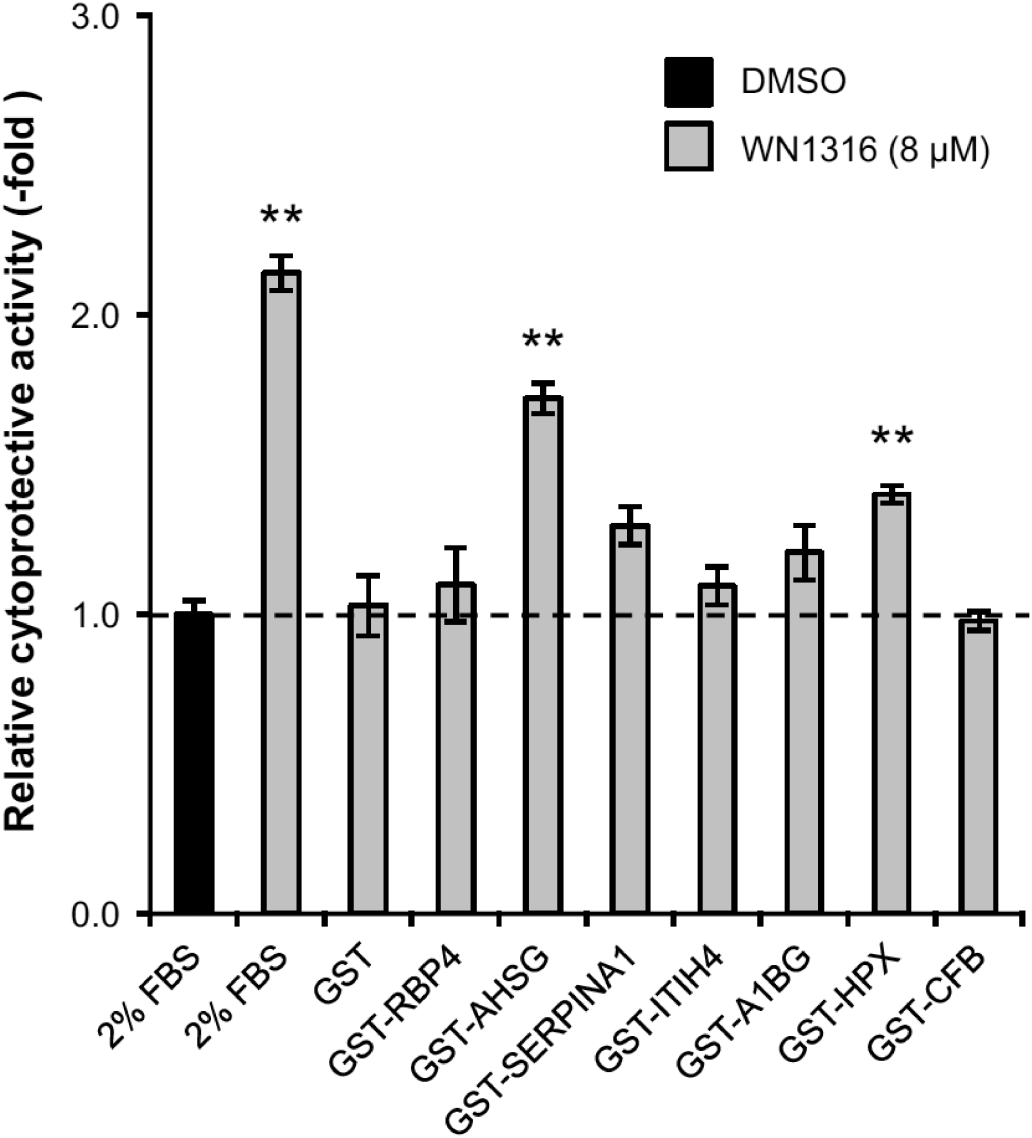
Human WN1316-activating factors exert WN1316-mediated cytoprotective activity. Differentiated SH-SY5Y cells were treated with 8 μM WN1316 in the presence of purified GST-fusion proteins for 3 h followed by 12 h of chase incubation without the compound, and then exposed to 40 μM menadione for 4 h. Concentrations of the GST-fusion proteins used are as follows; GST; 4.4 μg/ml, GST-RBP4; 2.2 μg/ml, GST-AHSG; 1.9 μg/ml, GST- SERPINA1; 2.4 μg/ml, GST-ITIH4; 2.3 μg/ml, GST-A1BG; 1.5 μg/ml, GST-HPX; 1.7 μg/ml, GST-CFB; 3.3 μg/ml. As control experiments, differentiated SH-SY5Y cells were treated with 8 μM WN1316 (as a positive control) or DMSO (as a vehicle control) in DMEM supplemented with 2% FBS (corresponds to a protein concentration of approximately 0.5 mg/ml). The cell viability was calculated by AlamarBlue, and was expressed as a relative value (relative cytoprotective activity; -fold) of the WN1316-treated samples for vehicle control (DMSO) set as 1. Data are expressed as mean ± SD (n = 4). Statistical significance was evaluated by one-way ANOVA (*p*<0.0001) followed by Dunnett’s *post hoc* test compared with GST-RBP4 (**p<0.001).

To identify the genuine WN1316-activating factor(s) from the synthesized candidate proteins, we performed AOND assay using differentiated SH-SY5Y cells. We confirmed no WN1316 cytoprotective effect both in RBP4 and GST (Fig 8). Among cell-free synthesized candidates, two proteins, AHSG and HPX, significantly exerted the WN1316 cytoprotective activity when compared to RBP4 (Fig 8). In particular, AHSG showed among the highest cytoprotective activity (1.7-fold) compared to the vehicle control.

Finally, to verify the WN1316 cytoprotective activity of TF, we carried out AOND assay in the presence of various concentrations of TF. The WN1316-mediated cytoprotective activity was significantly increased at extremely high concentrations of TF in a dose-dependent manner (S3 Fig). However, no significant cytoprotective activity was observed at lower TF levels, even though those concentrations of TF were still approximately 10 times higher than the effective concentration of either AHSG or HPX; 10–25 μg/ml for TF v.s. 1.7–1.9 μg/ml for AHSG or HPX. Therefore, we excluded TF from the candidate of WN1316-activating factor.

## Discussion

In this study, we attempted to unveil the biological WN1316-activating factors in FBS, and successfully identified nine candidate serum proteins by using a proteomic approach in conjunction with quantitative WN1316 cytoprotective activity analysis. Among them, two *N*-linked glycoproteins, alpha-2-HS-glycoprotein (AHSG) and hemopexin (HPX), markedly exhibited the WN1316-mediated neuroprotection against oxidative injury in a glycan-independent manner, indicating that they are endogenous factors implicating in small molecule compound-mediated neuroprotection. To our knowledge, this is a first report that shows concrete evidences for the novel glycan-independent function of serum glycoproteins in drug efficacy.

Novel amino imidazole derivative, WN1316, is a high blood-brain barrier (BBB) permeable small molecule compound that can alleviate disease progression via suppression of glial inflammation in ALS mouse models [2]. Two unexpected findings were obtained from our previous studies. First, the efficacy of WN1316 in ALS mouse models emerged is hundred times lower concentration than its potency in cultured human neuronal cells *in vitro* [2]. Second, FBS in culture media is a requisite for the cytoprotective activity of WN1316 *in vitro* (Fig 1). These findings combined led us to hypothesize certain serum factors being involved in exerting the WN1316-mediated anti-oxidative cell death activity. In this study, we have successfully identified two requisite serum factors, AHSG and HPX, in FBS for the WN1316-mediated cytoprotective activity, demonstrating direct proof for the existing of such factors. Additionally, the human homologues of the glycan free AHSG and HPX proteins synthesized *in vitro* worked as well.

Thus far, there has been no report showing the evidences that AHSG and HPX are directly and/or indirectly involved in neuroprotection in the CNS. AHSG, commonly referred to as fetuin-A, is a carrier plasma glycoprotein produced in the liver [17, 18]. AHSG is known to be a multi-functional protein and interacts with various proteins, such as transforming growth factor-β (TGF-β), bone morphogenetic protein (BMP), cytokines, and insulin receptor [19–25]. Among these functions, it is noteworthy that AHSG plays a role in membrane trafficking. Since AHSG binds to lipids and is internalized into cells via endocytosis [20–22], it is possible that endocytosis is the mechanism of action involving in WN1316 uptake into cells.

Other candidate protein, HPX, is a plasma glycoprotein with a high heme binding affinity, which is mainly produced in the liver but also in the CNS and the peripheral nervous system [26]. Interestingly, the heme-HPX complex is internalized via low-density lipoprotein receptor-related protein 1 (LRP1)-mediated endocytosis into cells [27]. Several studies have demonstrated that LRP1 is highly expressed in brain microvessels [28], and can transport several ligands across the BBB [29, 30]. Remarkably, it has been reported that the transmembrane LRP1 is expressed in SH-SY5Y cells, which were used in the present study, and plays a crucial role in the cytotoxicity of amyloid-β oligomers [31]. These findings suggest that the HPX molecules are, at least in part, implicated in delivering WN1316 into SH-SY5Y cells via the LRP1-mediated transport system.

One important question arising from this study is as to how AHSG and HPX deliver WN1316 into cells. Thus far, it has not yet been demonstrated whether WN1316 can form the complex with AHSG or HPX. However, since the WN1316-mediated cytoprotective activity is totally dependent on the presence of either purified AHSG or HPX, it is conceivable that these specific serum factors directly bind to WN1316 and act as carriers for transporting WN1316 to the target site within cells. Since the degree of WN1316-mediated activation by purified AHSG or HPX was lower than those by FBS, it is still possible that additional factors are needed to fully exert the WN1316-meidated activity. One alternative explanation is that some enzymatic activities present in FBS, which are mediated by factors other than AHSG and/or HPX, increase the WN1316-mediated cytoprotective activity by modifying the chemical structure of WN1316. However, since the HPLC-detected peak retention times of WN1316 in the brain tissues and serum were same as that in the purified compound [2], such metabolic effects might be marginal if existed. Nonetheless, when taken into account the fact that the effective concentration of WN1316 *in vitro* is extremely high when compared *in vivo* [2], it is reasonable that either other non-serum factors or serum factors are not present in FBS, or both of them play important roles in WN1316-mediated neuroprotection. Further investigations on the mode of interaction between WN1316 and AHSG or HPX, the route of the internalization of WN1316 mediated by AHSG and HPX, and the cooperative functional interactions of AHSG or HPX with other endogenous factors are required.

Another question arising from this study includes whether AHSG and HPX act specifically on WN1316. WN1316 is the only compound implicating in the serum factor-dependent cytoprotective function, which we have identified thus far. However, it does not necessarily mean that WN1316 is a sole amino imidazole derivative possessing such function, since we have not conducted a comprehensive screening of the compounds based on the AHSG/HPX-dependent cytoprotective activity yet. Future studies will uncover the relationship between the chemical structural feature of the small molecule compound and the AHSG/HPX-mediated activity.

At present, one of the most promising approaches for drug delivery to the brain is to utilize the receptor-mediated transcytosis (RMT) system [32–35]. Although it has been tried to utilize various receptors that are expressed in the BBB for the RMT system, the efficiency of CNS drug delivery via RMT depends on the expression level and distribution of target receptor in the BBB. Notably, utilization of ubiquitously expressed receptors for BBB transport causes incorrect delivery of CNS drug to other tissues and organs and could induce undesirable side effects [35, 36]. Therefore, discovery of the new BBB delivery route will give additional choices of delivery methods in terms of selectivity, capacity, and immune tolerance of CNS drugs. Although it is currently still unknown whether AHSG, HPX, and/or other factors are required to exert the WN1316-dependent neuroprotective activity *in vivo*, the administration of WN1316 can, indeed, alleviate the disease progression in ALS mouse models without any adverse side effects or complications [2]. It is plausible that the AHSG/HPX-mediated drug delivery could become an alternative means to treat the CNS diseases. Future pharmacological studies on WN1316 in conjunction with the use of AHSG/HPX conditional knocked-out animals will provide a novel alternative drug delivery pathway and the improvement of the cell membrane permeability of the CNS therapeutic agents.

In conclusions, we identified two glycoproteins, AHSG and HPX, that played a key role in the WN1316-mediated cytoprotective activity *in vitro*, suggesting that they could be ever-unidentified endogenous factors implicating in neuroprotective drug efficacy. Since WN1316 is a high BBB permeable small molecule compound and a promising therapeutic candidate for ALS [2], we have completed the first-in-Man clinical test of WN1316 and shown to be safe in healthy adult persons (unpublished data). To further proceed the clinical trials, it is extremely important to elucidate pharmacodynamics of WN1316 *in vivo* for its safer therapeutic application to the CNS diseases.

## Supporting information

S1 Fig Fractionation of WN1316-activating factor by HiTrap Blue column. Differentiated SH-SY5Y cells were pretreated with 8 μM WN1316 in DMEM containing 0.02 mg/ml Blue pass or Blue elute for 3 h followed by 12 h of chase incubation without the compound, and then exposed to 40 μM menadione for 4 h. As control experiments, differentiated SH-SY5Y cells were treated with 8 μM WN1316 (as a positive control) or DMSO (as a vehicle control) in DMEM supplemented with 2% FBS (corresponds to a protein concentration of approximately 0.5 mg/ml). The cell viability was calculated by AlamarBlue, and was expressed as a relative value (relative cytoprotective activity; -fold) of the WN1316-treated samples for vehicle control (DMSO) set as 1. Data are expressed as mean ± SD (n = 4). Statistical significance was evaluated by one-way ANOVA (*p*<0.0001) followed by Dunnett’s *post hoc* test compared with DMSO-treated control (**p<0.001). *Blue pass*, pooled proteins from pass-through fraction. *Blue elute*, pooled proteins from the column by a stepwise elution with 2 M NaCl.
(TIF)

S2 Fig Representative 2DE gel images of three types of elution after Bio-Scale Mini CHT Type I chromatography. 2DE pattern of (A) non-active fraction W1, (B) the highest-active fraction E1, and (C) low-active fraction C1 separated by the Bio-Scale Mini CHT Type I column. Gels were stained with SYPRO Ruby dye. The protein spots were classified into 5 groups.
(TIF)

S3 Fig Effect of transferrin on the WN1316-mediated cytoprotective activity. Differentiated SH-SY5Y cells were incubated with 8 μM WN1316 in serum free DMEM containing the indicated concentrations of transferrin (TF) for 3 h followed by 12 h of chase incubation without the compound, and then treated with 40 μM menadione for 4 h. As control experiments, differentiated SH-SY5Y cells were treated with 8 μM WN1316 (as a positive control) or DMSO (as a vehicle control) in DMEM supplemented with 2% FBS (corresponds to a protein concentration of approximately 0.5 mg/ml). Data are expressed as mean ± SD (n = 4). Statistical significance was evaluated by one-way ANOVA (*p*<0.0001) followed by Dunnett’s *post hoc* test compared with DMSO-treated control (**p<0.001, *p<0.01).
(TIF)
