# Supplementary figures and images for: A novel function of N-linked glycoproteins, alpha-2-HS-glycoprotein and hemopexin: Implications for small molecule compound-mediated neuroprotection
Source: PLoS One. 2017 Oct 9;12(10):e0186227. doi: 10.1371/journal.pone.0186227 (PMC5633190; doi:10.1371/journal.pone.0186227)

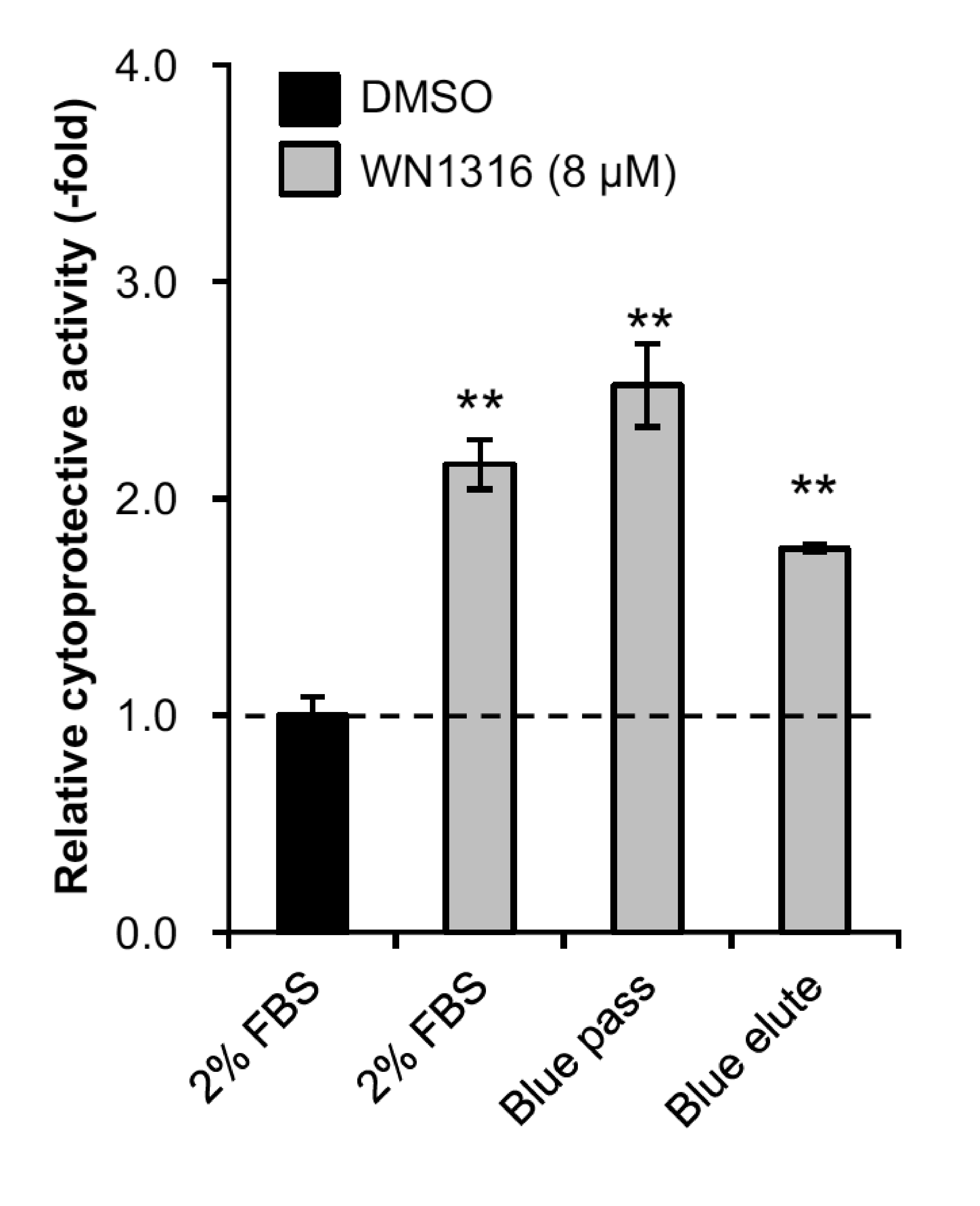

Supplement: S1 Fig — Differentiated SH-SY5Y cells were pretreated with 8 μM WN1316 in DMEM containing 0.02 mg/ml Blue pass or Blue elute for 3 h followed by 12 h of chase incubation without the compound, and then exposed to 40 μM menadione for 4 h. As control experiments, differentiated SH-SY5Y cells were treated with 8 μM WN1316 (as a positive control) or DMSO (as a vehicle control) in DMEM supplemented with 2% FBS (corresponds to a protein concentration of approximately 0.5 mg/ml). The cell viability was calculated by AlamarBlue, and was expressed as a relative value (relative cytoprotective activity; -fold) of the WN1316-treated samples for vehicle control (DMSO) set as 1. Data are expressed as mean ± SD (n = 4). Statistical significance was evaluated by one-way ANOVA (p<0.0001) followed by Dunnett’s post hoc test compared with DMSO-treated control (**p<0.001). Blue pass, pooled proteins from pass-through fraction. Blue elute, pooled proteins from the column by a stepwise elution with 2 M NaCl. (TIF) [file pone.0186227.s001.tif]

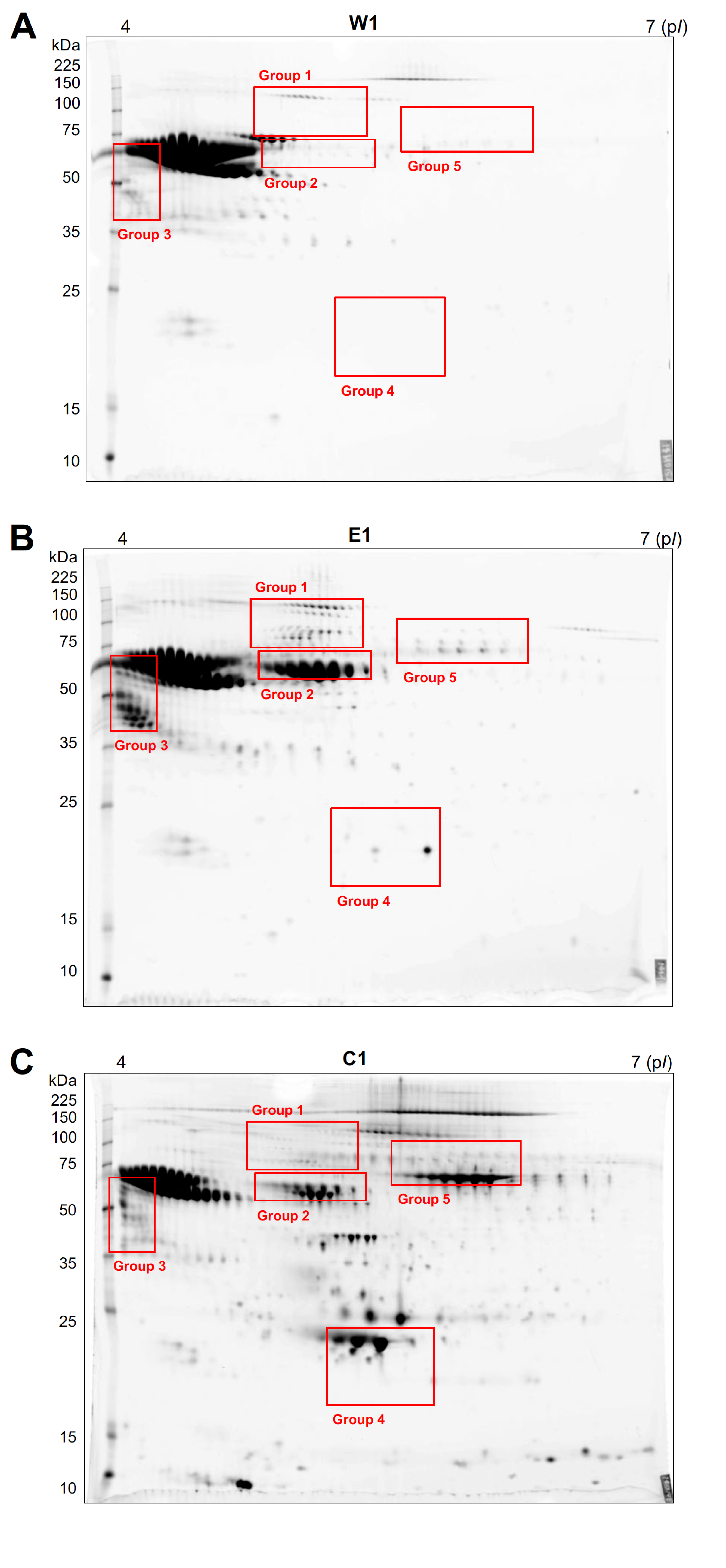

Supplement: S2 Fig — 2DE pattern of (A) non-active fraction W1, (B) the highest-active fraction E1, and (C) low-active fraction C1 separated by the Bio-Scale Mini CHT Type I column. Gels were stained with SYPRO Ruby dye. The protein spots were classified into 5 groups. (TIF) [file pone.0186227.s002.tif]

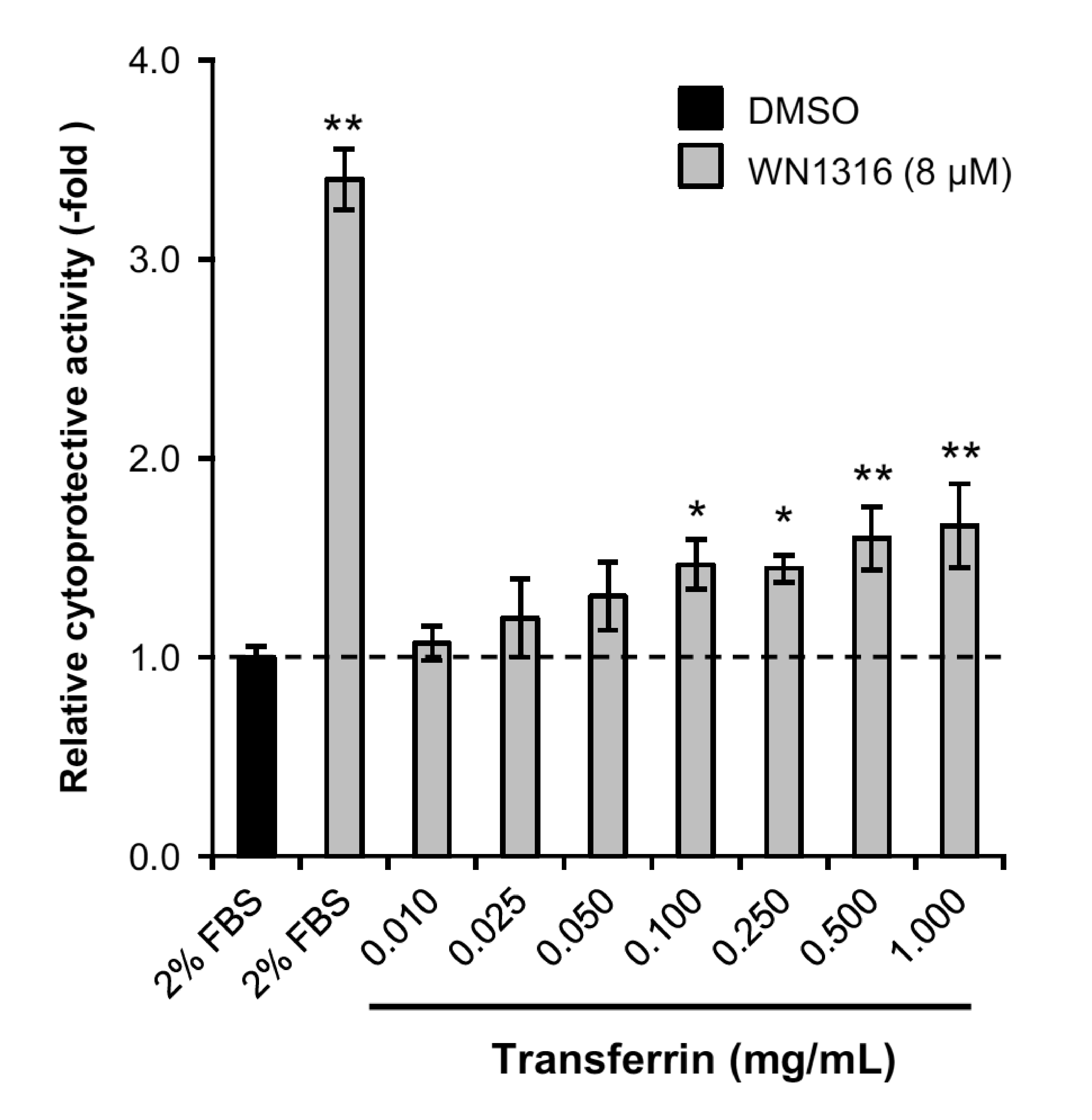

Supplement: S3 Fig — Differentiated SH-SY5Y cells were incubated with 8 μM WN1316 in serum free DMEM containing the indicated concentrations of transferrin (TF) for 3 h followed by 12 h of chase incubation without the compound, and then treated with 40 μM menadione for 4 h. As control experiments, differentiated SH-SY5Y cells were treated with 8 μM WN1316 (as a positive control) or DMSO (as a vehicle control) in DMEM supplemented with 2% FBS (corresponds to a protein concentration of approximately 0.5 mg/ml). Data are expressed as mean ± SD (n = 4). Statistical significance was evaluated by one-way ANOVA (p<0.0001) followed by Dunnett’s post hoc test compared with DMSO-treated control (**p<0.001, *p<0.01). (TIF) [file pone.0186227.s003.tif]
